# Symptom association between social anxiety disorder, appearance anxiety, and eating disorders among Chinese University students: A network analysis to conceptualize comorbidity

**DOI:** 10.3389/fpubh.2022.1044081

**Published:** 2022-12-22

**Authors:** Yu Jin, Shicun Xu, Chang Chen, Amanda Wilson, Desheng Gao, Yan Ji, Xi Sun, Yuanyuan Wang

**Affiliations:** ^1^College of Education for the Future, Beijing Normal University, Zhuhai, China; ^2^Northeast Asian Research Center, Jilin University, Changchun, China; ^3^Department of Population, Resources and Environment, Northeast Asian Studies College, Jilin University, Changchun, China; ^4^China Center for Aging Studies and Social-Economic Development, Jilin University, Changchun, China; ^5^Key Laboratory of Brain, Cognition and Education Sciences, Ministry of Education, South China Normal University, Guangzhou, China; ^6^Guangdong Key Laboratory of Mental Health and Cognitive Science, School of Psychology, Center for Studies of Psychological Application, South China Normal University, Guangzhou, China; ^7^Division of Psychology, Faculty of Health and Life Sciences, De Montfort University, Leicester, United Kingdom; ^8^School of Marxism, Harbin Institute of Technology, Harbin, China

**Keywords:** social anxiety disorder, appearance anxiety, eating disorder, network analysis, young adults

## Abstract

**Background:**

Incidences of social anxiety disorder (SAD), appearance anxiety, and eating disorders (ED) show an increased prevalence among young people. However, symptoms' associations between these disorders have not been investigated in depth. Network analysis is an approach that can be used to explain the relationship(s) between symptoms of different psychological disorders. Using network analysis, this study aimed to explore the association and potential interacting mechanisms between SAD, appearance anxiety, and ED.

**Methods:**

This study included 96,218 University students from Jilin Province, China. SAD, appearance anxiety and ED were assessed using the Social Anxiety Subscale of the Self-Consciousness Scale (SASS), the Appearance Anxiety Scale Brief Version (AASBV), and the Sick, Control, One, Fat, Food questionnaire (SCOFF), Chinese versions. Network analysis was employed to investigate the symptom associations, while the stability of the network model was analyzed using statistical measures.

**Results:**

The prevalence of ED among the total sample was 38% (95% CI: 38.1–38.8%), while this figure was 31.2% (95% CI: 30.7–31.6%) in males and 43.6% (95% CI: 43.2–44.0%) in females. Additionally, the total score of SAD was significantly higher in females (11.83 ± 5.37) than it in males (10.02 ± 5.46) (*P* < 0.001). While the total score of appearance anxiety was also different significantly in gender (39.21 ± 9.49 in females vs. 38 ± 9.42 in males) (*P* < 0.001). Results showed that ED was associated with all three aspects of appearance anxiety, including “appearance concern,” “appearance satisfaction,” and “wish for good looks.” Appearance anxiety and SAD were also associated; specifically, symptoms of “appearance satisfaction” were significantly associated with the symptoms “easily talk to strangers” and “appearance concern”, which was also significantly associated with “embarrassed”. Compared with males, females showed significantly stronger associations with appearance anxiety symptoms, while ED symptoms were associated with “troubled by being watched” and “appearance concern”.

**Conclusion:**

Appearance anxiety was associated with both ED and SAD symptoms. ED may have a potential relationship with SAD, affecting appearance anxiety indirectly. Significant differences were found among males and females in symptom associations between appearance anxiety and SAD. This study therefore clarified that young people should have body-positive interventions and challenge the normative body image discourse, which may help alleviate symptoms of SAD and ED.

## 1. Introduction

Social anxiety disorder (SAD), also known as social phobia, is a persistent fear of being exposed to strangers in one or more social or professional situations, where people might feel they are being observed by others, and is marked by a fear of engaging in embarrassing situations or behaviors (1, 2). SAD is common in young people and can severely impair their social functioning (3). The onset of SAD is around 15.5 years old, with the prevalence being between 4.0–8.7% among 14 and 24 years old (4, 5). Young people with SAD often experience excessive or unreasonable anxiety when exposed to social or performance-based stimuli (6). Studies have shown that SAD is often accompanied by extreme attention to appearance and frequently repeated observations of appearance (7). People with SAD have deficits of self-cognition and tend to criticize their own social abilities and physical appearance, resulting in problems such as appearance anxiety (8).

Individuals with SAD are prone to negative self-evaluation when it comes to their appearance; this deficit in perception of appearance can also exacerbate poor body image development (9–11). Appearance anxiety refers to individuals who pay excessive attention to their appearance due to perceived social standards and perceptions of other people's evaluations, which consequently generate insecurity, doubt, and anxiety about their appearance (12). Several studies have suggested that appearance anxiety can positively predict SAD among normal samples, and even among eating disorder samples (8, 13, 14). In turn, SAD symptoms also predict appearance anxiety and other body image disorders (13, 15). Furthermore, appearance anxiety is highly correlated with eating disorders such as anorexia nervosa and bulimia nervosa (16), and appearance-related comments can increase appearance anxiety and directly or indirectly affect eating disorders (17–19). Eating disorders (ED) are a group of psychiatric disorders characterized by abnormal eating behaviors and emotional disturbances, accompanied by significant weight changes and symptoms of physiological dysfunction syndrome (20). Previous studies have proposed there is an association between appearance anxiety and ED. For example, Li found that appearance anxiety positively predicted ED in 2,509 high school students (21). Levinson and Rodebaugh demonstrated that appearance anxiety symptoms predicted ED symptoms up to 6 months later and vice versa (22). In addition, ED may be closely related to anxiety disorders and thus may lead to negative emotions and interpersonal difficulties among individuals (23). Fredrickson and Roberts (24) noted that individuals might pander to or resist social appearance comments, subsequently increasing their maladaptive ED behaviors. This suggests that those affected by the ideal body shape promoted within a society may display negative emotions related to social appearance anxiety, and then develop maladaptive reactions and restrictions, including EDs (25, 26).

Although appearance anxiety is a common risk factor for SAD and ED, the overall mechanism of action of the three remains to be explored. For instance, difficulty in public eating is a bridge symptom between ED and SAD, with appearance anxiety playing a transitional role (27). Thus, there is a potential association between SAD and ED symptoms as expressed through appearance anxiety. Research also indicates a high comorbidity between SAD and ED (8). According to the results from the literature reviewed, the comorbidity rate of SAD and ED is 68% (28). Moreover, appearance anxiety may increase the risk of ED and SAD (14, 29). Research further points to the increased risk of appearance anxiety in ED patients (16), and that appearance-related comments can increase appearance anxiety, directly or indirectly affecting ED (17–19). However, the potential symptoms' associations between ED, SAD and appearance anxiety still require additional exploration.

Network analysis is an approach that can explain the symptoms individuals experience as part of their diagnosis. The method can explore the complexity of psychological symptoms and focus on the adjustment of dynamic feedback between different symptom items on a scale (30–32). In the network model, symptoms of the disorders are defined as nodes, while associations between symptoms are defined as edges (33). When there are significantly strong associations between symptoms in two disorders, these specific symptoms would be described as bridge symptoms. These bridge symptoms result in a transformation from one disorder to another, playing an essential role in maintaining the network model, which occurs simultaneously. Therefore, as a visualized method, network analysis can identify strong links between symptoms of different psychological disorders and provide evidence for pathogenesis research (34, 35). For example, among young people with childhood sexual abuse experience, associations between psychosis and post-traumatic stress disorder (PTSD) symptoms include hypervigilance, intrusive thoughts, and physiological and emotional reactivity (36). Other studies also found connections between depression and anxiety (37), anxiety and insomnia (38), and other mental health disorder symptoms (39, 40). The discovery of bridge (joining) symptoms can improve the efficiency of monitoring young people and help provide targeted interventions among young people with psychological disorders (40, 41).

Thus, network analysis is applied to investigate the following complexities: first, to explore the symptom association between appearance anxiety, SAD, and ED as three separate groups. Second, to explore the potential relationship between ED and SAD, and the role of appearance anxiety as the mechanism of action. More comprehensive guidance and targeted interventions need to provide for patients with appearance anxiety, SAD, and ED to improve their quality of life.

## 2. Methods

### 2.1. Study design

From October to November 2021, an online questionnaire was distributed to students in 63 Universities in Jilin province, China. Cross-sectional questionnaires were collected from 117,248 students. All participants signed an online informed consent form before answering the online questionnaire. The Ethics Committee of Jilin University approved this study.

### 2.2. Measurements

#### 2.2.1. Appearance anxiety

Appearance anxiety was measured by the Appearance Anxiety Scale Brief Version (AASBV). The Appearance Anxiety Scale has 14 items in the abbreviated version (12). Items are scored on a Likert scale of 1 (not at all) to 5 (very good)—the total score of this subscale ranges from 14 to 70 points. The three factors of the scale include: “appearance concern,” “appearance satisfaction,” and “wish for good looks” (42, 43). The Chinese version of the Appearance Anxiety Scale Brief Version (AASBV) has reliable internal consistency, with a Cronbach's alpha of 0.83 in females and 0.78 in males (44).

#### 2.2.2. Social anxiety disorder

The Social Anxiety Subscale of the Self-Consciousness Scale (SASS) has six items and uses a 5-point Likert scale. The total score ranges from 0 to 24 points (45). The higher the total score, the more severe the level of social anxiety. This scale measures subjective anxiety, verbal, and behavioral difficulties. The Chinese version has good reliability and validity, with a Cronbach's alpha of 0.72 (46).

#### 2.2.3. Eating disorder

The Sick, Control, One, Fat, Food (SCOFF) questionnaire contains five characteristics of eating disorders: Sick, Control, One, Fat, and Food (47). A score of 0 means no, and 1 means yes. The total score of this subscale ranges from 0 to 5 points. The scale had a sensitivity of 73.1% and a specificity of 77.7% for ED (47). Scale scores with more than two points were considered as displaying ED tendencies.

### 2.3. Statistical analysis

#### 2.3.1. Network estimation

Partial correlation networks were used to assess the association between appearance anxiety, SAD, and ED among participants. The Graphical Gaussian Model (GGM) was applied to build the network model. Then the graphic least absolute shrinkage and selection operator (LASSO) algorithm was used to perform a sparse network model by deleting unimportant associations. This approach was performed using the R package “qgraph” (48, 49). For each node, excepted influence (EI) represents the summed weight of all its edges, positive and negative, with its immediate neighbor nodes in the network (53). Predictability indicates how much variation in a node can be predicted by variation in the nodes connected to it. If a node has a high degree of centrality and predictability, this lends credence to the interpretation of its network importance.

#### 2.3.2. Network accuracy and stability

To examine the robustness of results, three procedures were performed. First, the accuracy of edge-weights was determined using the non-parametric bootstrapping approach to compute confidence intervals (CIs) (51). The main dataset was then randomly re-sampled to generate additional data points from which the 95% CIs were determined. Second, using subset bootstraps, the correlation stability coefficient (CS-C) was determined to examine the stability of the EI centrality (48, 52). Finally, bootstrapped difference tests were used to assess differences in the attributes of the network (53).

#### 2.3.3. Network comparison

The Network Comparison Test (NCT) was performed to examine statistical differences among the networks between the males and females. This methodology assesses differences in network structure, global strength and each edge between the two networks using Holm-Bonferroni correction (50). The significant edge differences for each pair of the groups were plotted after statistical testing. These tests were analyzed using the R-package “NetworkComparisonTest” (54).

## 3. Results

### 3.1. Study sample

A total of 96,218 young people met the inclusion criteria and answered all the required questions included in the analysis to be included in the data set; more than half of them were young females (58.4%) and lived in urban cities (50.9%). The prevalence of ED among the total sample was 38% (95% CI: 38.1–38.8%), this figure was 31.2% (95% CI: 30.7–31.6%) in males and 43.6% (95% CI: 43.2–44.0%) in females. Additionally, the total score of SAD was significantly higher in females (11.83 ± 5.37) than in males (10.02 ± 5.46) (*P* < 0.001). While the total score of appearance anxiety was also different significantly in sex (39.21 ± 9.49 in females vs. 38 ± 9.42 in males) (*P* < 0.001). Significant differences in sociodemographic variables between males and females are shown in Table 1. The items of the three scales are displayed in Supplementary Table S1.

**Table 1:** Descriptive statistical analysis.

**Variables**	**Total (*N* = 96,218)**	**Male (*N* = 40,065)**	**Female (*N* = 56,153)**	***x*^2^/*T***	***P*-value**
**Residence**					
City	48,932 (50.9)	19,804 (49.4)	29,128 (51.9)	55.83	<0.001
Town and county	47,286 (49.1)	20,261 (50.6)	27,026 (48.1)		
**Ethnic**					
Han	86,111 (89.5)	36,257 (90.5)	49,855 (88.8)	72.99	<0.001
Others	10,107 (10.5)	3,808 (9.5)	6,299 (11.2)		
**Family type**					
Nuclear family	66,888 (69.5)	27,259 (68.0)	39,630 (70.6)	300.2	<0.001
More than three generation	17,622 (18.3)	8,320 (20.8)	9,302 (16.6)		
Others	11,708 (12.2)	4,486 (11.2)	7,222 (12.8)		
**Current annual income**					
Under ¥6,000	28,624 (29.8)	11,284 (28.2)	17,341 (30.9)	233	<0.001
¥6,000–13,999	31,212 (32.4)	12,677 (31.6)	18,535 (33)		
¥14,000–22,999	16,061 (16.7)	6,752 (16.9)	9,309 (16.6)		
¥23,000 and above	20,321 (21.1)	9,352 (23.3)	11,069 (19.7)		
**Only-child**					
Yes	45,660 (47.5)	22,349 (55.8)	23,311 (41.5)	1,909	<0.001
No	50,558 (52.6)	17,716 (44.2)	32,843 (58.5)		
Age	19.59 ± 1.74	19.64 ± 1.80	19.56 ± 1.70	6.52	<0.001
SCOFF	1.31 ± 1.33	1.1 ± 1.29	1.46 ± 1.33	−42.3	<0.001
SASS	11.08 ± 5.48	10.02 ± 5.46	11.83 ± 5.37	−51.3	<0.001
AASBV	38.71 ± 9.42	38 ± 9.28	39.21 ± 9.49	−19.6	<0.001

SCOFF, the Sick, Control, One, Fat, Food questionnaire; SASS, the Social Anxiety Subscale of the Self-Consciousness Scale; AASBV, Appearance Anxiety Scale Brief Version.

### 3.2. Network estimation between symptoms of ED, appearance anxiety, and social anxiety

**Figure 1:**
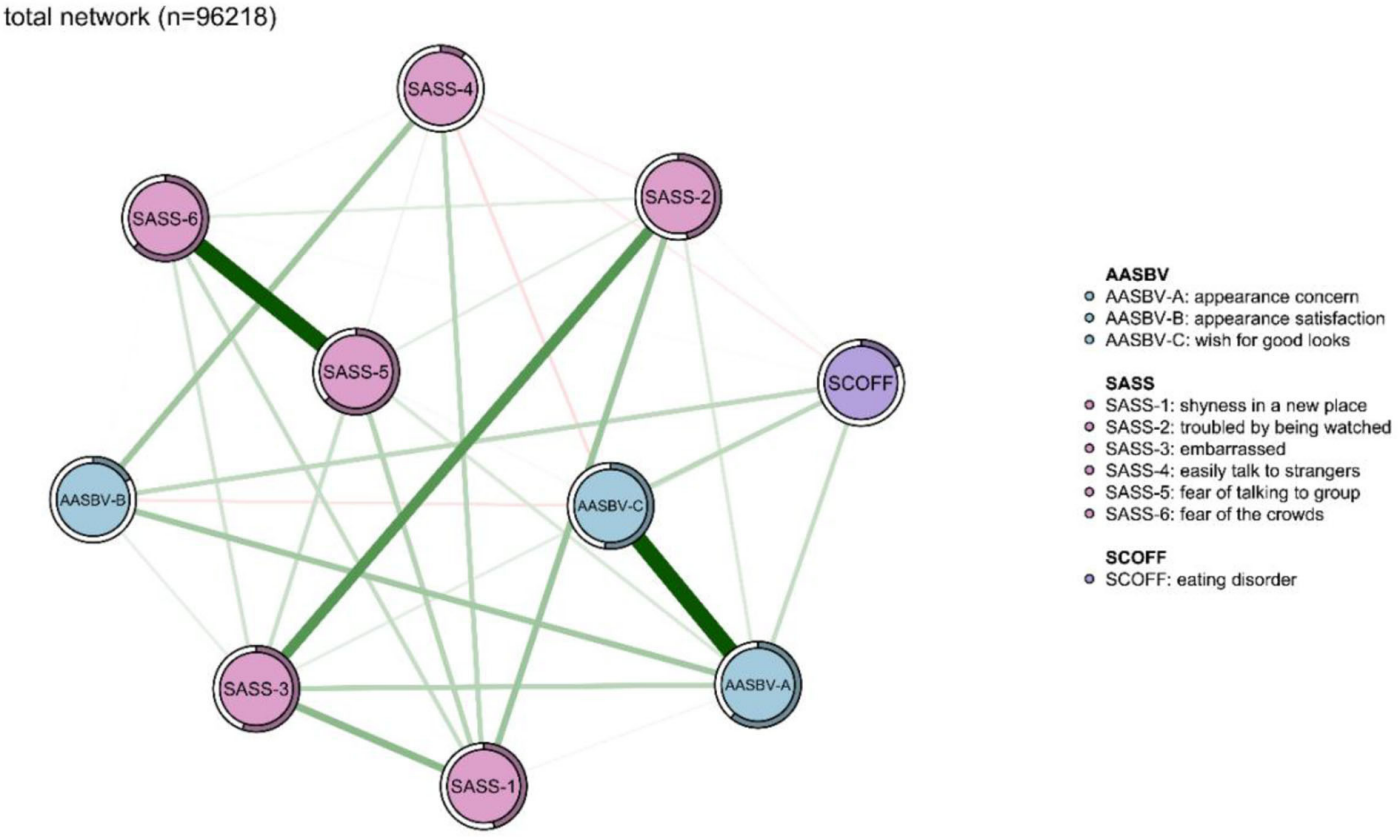
Network analysis between symptoms of eating disorder, appearance anxiety, and social anxiety in the total sample (*n* = 96,218). The thickness of the edges indicates the magnitude of the association. Green edges referred to positive associations, while red edges showed negative ones. The circle around the item represents the amount of predictivity.

**Figure 2:**
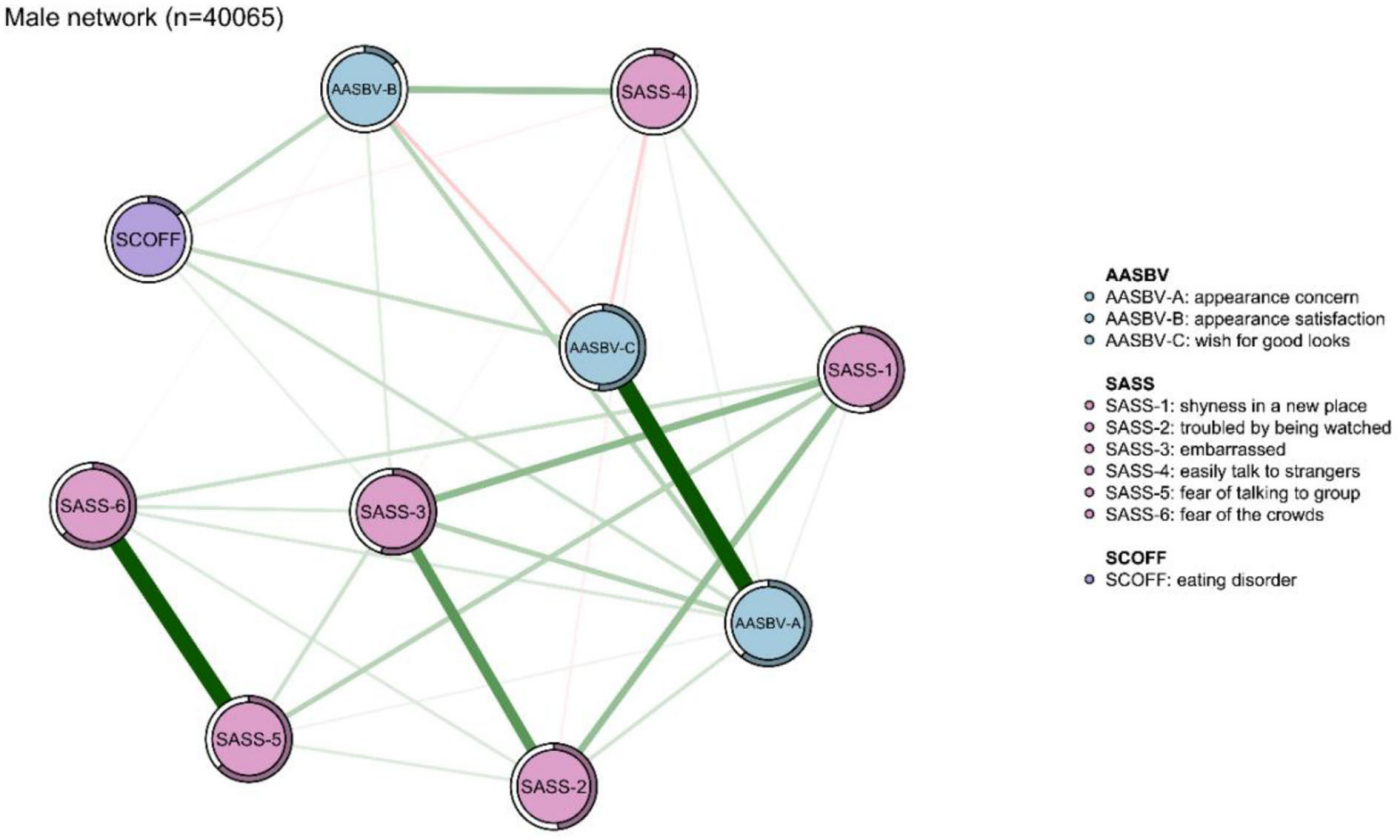
Network analysis between symptoms of eating disorder (ED), appearance anxiety, and social anxiety disorder (SAD) in the male sample (*n* = 40,065). The thickness of the edges indicates the magnitude of the association. Green edges referred to positive associations, while red edges showed negative ones. The circle around the item represents the amount of predictivity.

**Figure 3:**
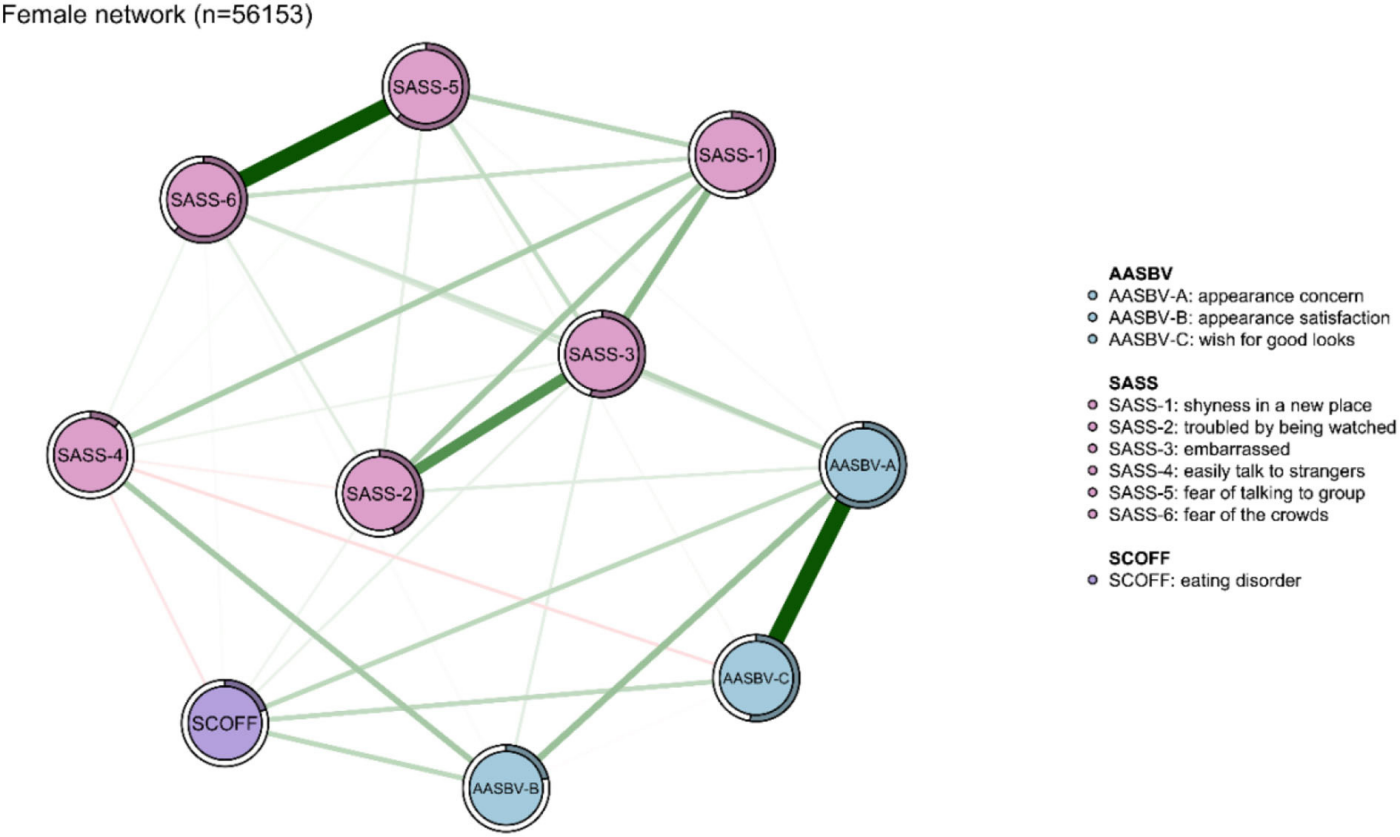
Network analysis between symptoms of eating disorder, appearance anxiety, and social anxiety in the female sample (*n* = 56,153). The thickness of the edges indicates the magnitude of the association. Green edges referred to positive associations, while red edges showed negative ones. The circle around the item represents the amount of predictivity.

The network models among the total sample, as well as the network models for both male and female participants, are presented in Figures 1, 2. As shown in Figures 2, 3, network structures in males and females were similar to the structure of the total population. In the total sample, as seen in Figure 1, several items in the same scale were connected strongly, such as “fear of the crowds” connected to “fear of talking to group”, and “troubled by being watched” connected to “embarrassed”, among those with SAD. The SAD items were significantly associated with appearance anxiety, for example, “appearance satisfaction” was significantly associated with “easily talk to strangers”, and “appearance concern” was significantly associated with “embarrassed”. “Appearance satisfaction” and “appearance concern” were connected to appearance anxiety. ED was associated with all three aspects of appearance anxiety including “appearance concern,” “appearance satisfaction,” and “wish for good looks.” ED was also connected with SAD indirectly by affecting appearance anxiety symptoms. Predictability estimations are displayed in the network analysis (Supplementary Table S2). “Fear of talking to group,” “fear of the crowds,” and the “appearance concern” nodes were best explained by the associated nodes in the three groups. This result indicated that the three symptoms were therefore more likely affected by other connected symptoms.

### 3.3. Network accuracy and centrality

As measured by non-parametric CIs, the precision of edges was found to be satisfactory, with lower CIs suggesting more accurate edge estimates (Supplementary Figure S1). A considerable number of edge weight comparisons were statistically significant, according to the bootstrapped difference tests (Supplementary Figure S3). Even when a large portion of the sample was dropped, the betweenness, closeness, and strength values remained steady using the case-dropping subset bootstrap approach (Supplementary Figure S2). Strength, betweenness, and closeness showed an excellent level of stability (CS-C = 0.75) among the three groups (SAD, appearance anxiety, ED).

### 3.4. Network comparison results

**Figure 4:**
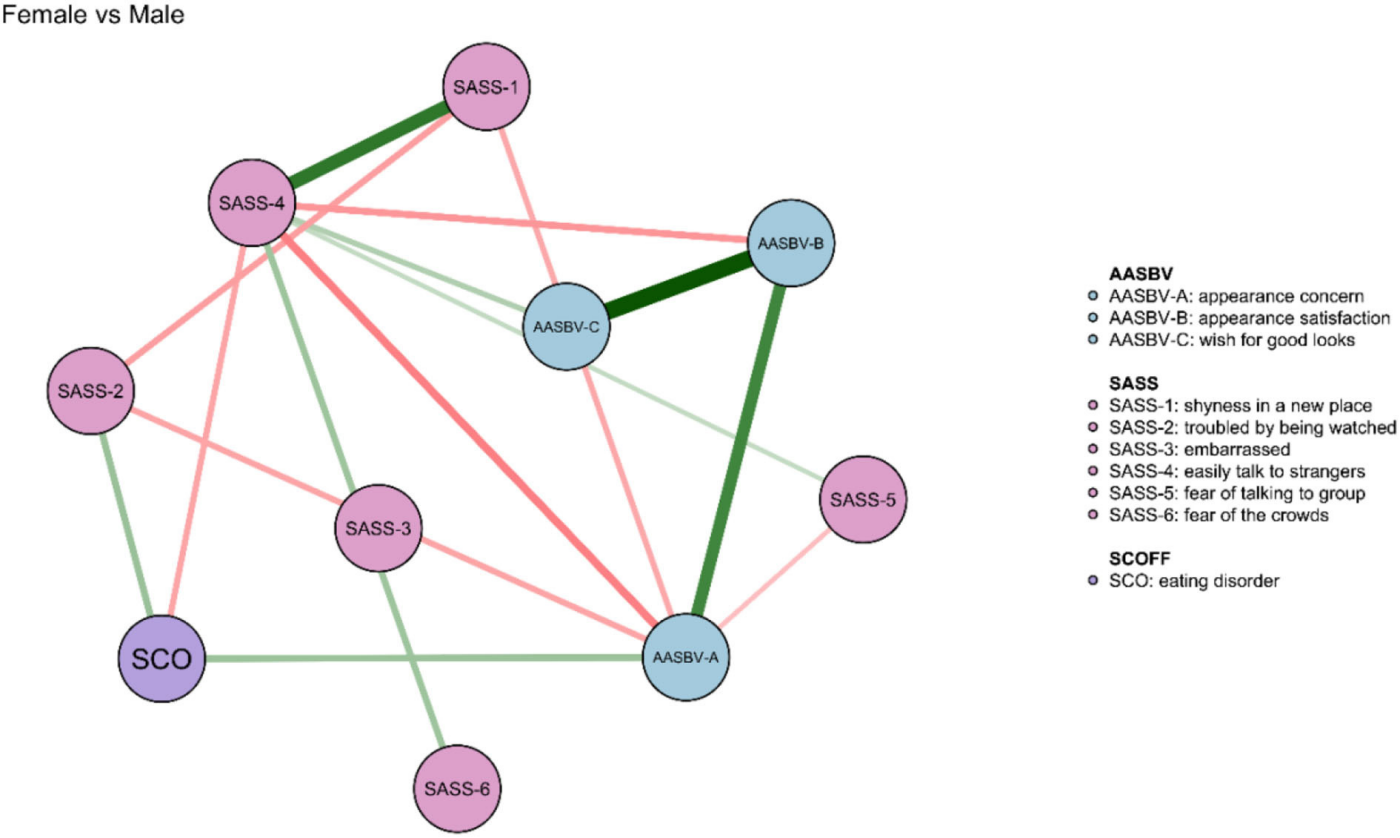
Comparison of symptom network model between the male and female sample. The green edges denote the stronger connections between symptoms in females when compared with males.

The comparison of network models between males and females was conducted. There was no significant difference in network global strength (*p* = 0.45) between the sexes. However, there was a significant difference between males and females in edge weights (*p* < 0.001). Figure 4 shows the significant differences in edge weights between males and females and the significant differences are described as follows. Compared with males, females showed stronger associations between “troubled by being watched” and “appearance concern”, between “wish for good looks” and “easily talk to strangers”, and finally between ED and “troubled by being watched”. Compared with females, males showed stronger associations between “easily talk to strangers” and ”appearance concern”, between “easily talk to strangers” and “appearance satisfaction”, and between ED and “easily talk to strangers”.

## 4. Discussion

This is the first study to use network analysis to explore symptom associations and potential mechanisms between SAD, ED, and appearance anxiety in a large sample of 96,218 in Jilin Province, China. The results among the three groups (total population, male, and female participants) showed that appearance anxiety was associated with both ED and SAD. The result indicates that there might be a potential relationship between ED and SAD by affecting the mechanism of appearance anxiety indirectly in young people. Furthermore, there are significant differences between the total scores of these three disorders, as well as the symptom associations between appearance anxiety, ED, and SAD in male and female participants.

The results of this study showed that ED was associated with all three aspects of appearance anxiety, including “appearance concern,” “appearance satisfaction,” and “wish for good looks.” These results were consistent with previous studies. For example, a study demonstrated a moderate correlation between appearance anxiety and ED, and the mediating effect of appearance anxiety on body esteem and ED in a structural equation model (21). Another study found that among 518 American University students, which measured sociocultural attitudes toward appearance, these attitudes significantly predicted ED in male students (55). Levinson and Rodebaugh examined the interaction between appearance anxiety and ED using follow-up studies 6 months apart, finding that appearance anxiety positively predicted ED and vice versa (22). These results could be explained by various other studies. First, University students were found to be more susceptible to social media perfection/pressures. This might lead them to develop a tendency to compare their appearance to others' perfect appearance, with negative self-appearance perceptions and appearance anxiety arising as a result of the comparison. They are more likely to develop ED to conform to social perfection (56). Second, fear of negative evaluation(s) may also be one of the reasons for the close relationship between appearance anxiety and ED symptoms (57). Third, studies have also suggested that individualized social ideals of appearance aggravate the link between appearance anxiety and ED (58). Similarly, and finally, negative emotions resulting from the comparison of actual and the perceived socially ideal appearance would increase the individual's negative perceptions of appearance and somatic disgust, thereby inducing ED-based symptoms. Since in our study appearance anxiety symptoms were closely related to ED symptoms, improving appearance anxiety might effectively reduce ED symptoms. Therefore, these results would help researchers develop interventions for ED patients to reduce appearance anxiety. Furthermore, positive emotions such as increased bodily appreciation might improve intuitive eating and promote relief of ED symptoms (59).

Symptom associations of appearance anxiety and SAD included connections between “appearance satisfaction” and “easily talk to strangers”, “appearance concern” and “embarrassed”, and “wish for good looks” and “easily talk to strangers”. These findings were also consistent with previous studies. In a 2-year follow-up study, it was reported that SAD was associated with the frequency of appearance comparisons, self-perceived attractiveness, and social media comparisons. Furthermore, appearance comparison behavior significantly predicted SAD 1 year later (60). Another study showed that self-expression, willingness to communicate and non-verbal communication were all significantly related to appearance anxiety (61). This significant relationship was also supported in the results of the current study. Moreover, a study further confirmed that negative evaluation of appearance is closely related to SAD symptoms and confirmed the positive mutual prediction between the two disorders (8).

On the other hand, the results showed that individuals who do not value appearance might be more likely to converse with strangers. Actually, if an individual worries too much about their appearance, it is possible to cause embarrassment in interpersonal communication. This phenomenon could be explained from the following perspectives. University is a prominent time of increased attention to appearance while the individual develops across the life course (62). Young adults with SAD are more likely to make inappropriate appearance comparisons, such as comparing themselves to a socially perfect image, which might exacerbate negative perceptions of appearance (63). In addition, individuals with SAD might have a vaguer self-concept, more negative cognitions about self-appearance, and quickly form the belief that others are more attractive than themselves (64, 65). This might also explain the results in this study, where symptoms of concern about appearance were more likely to be associated with symptoms of social embarrassment. A study also pointed out that positive peer relationships can negatively predict an individual's appearance comparison behaviors (64). Peer relationships are particularly important in the process of individual adolescent cognition and the formation of the basic social system; it is also one of the critical factors in the development of SAD (66). Difficulties with peer relationships might maintain and further develop negative appearance perceptions in individuals with SAD (67). The results of the present study further clarified the association between appearance anxiety symptoms and SAD symptoms. This is significant for guiding young people to establish a positive appearance that is not based on comparison. University students might rebuild their peers' social confidence if they develop a body positive perception discourse of self-appearance. All of these factors contributed to the prevention and intervention of SAD among young adults. The results of this study confirmed that ED might have a potential relationship with SAD by affecting appearance anxiety indirectly. Appearance anxiety was a common risk factor for SAD and ED. Previous studies also confirmed this result. For example, Levinson et al. (22, 27) found that appearance anxiety was a bridge symptom between SAD and ED. Positive prediction of appearance anxiety in SAD and ED also have been suggested in a previous study (8). The findings could also help patients with SAD and ED comorbidities by conducting cognitive behavioral therapy or virtual reality exposure therapy (9, 68).

There were also significant differences in the total scores, as well as the symptom associations between appearance anxiety, ED, and SAD in male and female participants. Compared with males, females showed significantly stronger associations between appearance anxiety symptoms, as well as ED with “troubled by being watched” and “appearance concern”. Previous studies also have reported that females are more likely to be concerned with the importance of their appearance than males (55, 60, 69). For instance, a study found that SAD and appearance anxiety were significantly higher in females than in males (60). Women also tend to make more physical comparisons than men (70). When individuals suffers from appearance anxiety, including body image dissatisfaction and concerns about shape and weight, they are prone to have ED symptoms concurrently (29). Moreover, the associations between body dissatisfaction, having positive feeling about thinness, and ED have also been established (71, 72). In actuality, both males and females are under significant pressure from sociocultural factors, including messages from family, peers, the media, and the general public. These factors may be crucial in determining how males and females perceive themselves in terms of their physical attractiveness and beauty. Furthermore, because of the social acceptability signals that are shown in society and the media to form the ideal body image, women also consequently equate physical appearance—often in the form of thinness—with success, power, and pleasure (73). In order to maintain a certain social standing and to compete for societal advantages, it is therefore believed that the propensity for dieting and the desire to reduce weight are important values for females (74, 75). On the other hand, dissatisfaction in appearance anxiety is not merely the result of an individual's personal dissatisfaction with their body image, but is also influenced by the fear of negative evaluation by others. University women are the most susceptible population to negative evaluation by others. For example, in comparison to University males, University females are more affected by parental comments on their body image (76). Slater and Tiggemann (42, 77) also found that appearance-related comments can lead to appearance anxiety in females. Significant gaps between actual and ideal types could lead to negative emotions, body shame, and anxiety in women, all of which are risk factors for psychiatric disorders such as ED (78–80). All these factors might explain the more serious symptoms of appearance anxiety and ED in females compared with males.

This study explored the association of symptoms between SAD, ED, and appearance anxiety through network analysis. It was determined that ED might have a potential relationship with SAD by affecting appearance anxiety indirectly. Moreover, the dynamic relationship between SAD and appearance anxiety symptoms also revealed the maintenance and development of ED symptomology. Our results also provide more detailed information for interventions for the related psychological disorders. The possible development of interventions and symptoms of the disorders discussed, provide the basis for the mechanism of action in this research. This study has limitations to note. First, the study design was a cross-sectional study. Therefore, the causal relationship between ED, SAD, and appearance anxiety may not be representative. Second, this study mainly focuses on the interaction between SAD, ED, and appearance anxiety. The researchers did not measure all the potential variables that may affect these disorders, such as depression and self-loathing as found by Szymanski and Henning (81), as well as appearance anxiety also affecting an individual's level of depression. While appearance anxiety was found to be associated with more incredible self-loathing in individuals (14), other variables, such as differences between urban and rural China and being an only child, could be examined in future research. Third, ED, SAD, and appearance anxiety were measured using self-report scales online, which may have resulted in recall bias. However, in order to avoid the impact of the pandemic, sending questionnaires online allowed for health and safety measures, and has been proven to be an effective method of recruitment (40). Moreover, while the Jilin Province was not in lockdown during the period of study, there still could have been an effect of COVID-19, particularly symptoms of anxiety which might bias the results of this study, inflating the resulting symptomology. Fourth, appearance anxiety can also exacerbate neurological symptoms (82). Therefore, in future research, neurological measurement should be added to explore relationships between appearance anxiety and neurological functioning. Finally, measures of body positivity should be used in the future to begin understanding the feasibility of creating an intervention for appearance anxiety.

## 5. Conclusion

Appearance anxiety was associated with both ED and SAD. ED may have a potential relationship with SAD by affecting appearance anxiety indirectly. Significant differences in symptom associations between appearance anxiety and SAD were further found among males and females. Females showed stronger associations between when being watched, worrying about their appearance, and symptoms of ED, and they tended to expect to be more attractive to reduce anxiety when talking to other people. The results of this study can help to guide the formation of body-positive interventions for young people and also provide additional ideas for intervening with SAD and ED.

## Data availability statement

The original contributions presented in the study are included in the article/Supplementary material, further inquiries can be directed to the corresponding authors.

## Ethics statement

The study involving human participants was reviewed and approved by Jilin University. The patients/participants provided their written informed consent to participate in this study.

## Author contributions

YaJ, YW, and SX: study design. SX, DG, YaJ, and XS: data collection and methodology. YuJ, YW, SX, AW, and CC: manuscript writing. All authors contributed to the article and approved the submitted version.
