# Supplementary material for: Symptom association between social anxiety disorder, appearance anxiety, and eating disorders among Chinese University students: A network analysis to conceptualize comorbidity
Source: Front Public Health. 2022 Dec 22;10:1044081. doi: 10.3389/fpubh.2022.1044081 (PMC9814491; doi:10.3389/fpubh.2022.1044081)
Supplement: Supplementary file 1 [file Data_Sheet_1.docx]

Supplementary masteries.

Table S1. Items of SCOFF, SASS and AASBV.

Table S1. Predictability of each item among three groups.

Figure S1. Bootstrapped confidence intervals of edge weights among three groups

Figure S2. Stability of centrality indices by case dropping subset bootstrap

Figure S3. Estimation of "ExpectedInfluence" by bootstrapped difference test among three groups.

Figure S4. The comparison of network models between males and females.

Table S1. Items of SCOFF, SASS, and AASBV.

| **Scale** | **Variable** | **Items** |
| --- | --- | --- |
| SCOFF | SCOFF-1 | Do you make yourself sick because you feel uncomfortably full? |
|  | SCOFF-2 | Do you worry you have lost control over how much you eat? |
|  | SCOFF-3 | Have you recently lost more than 14 pounds (one stone) in a 3-month period? |
|  | SCOFF-4 | Do you believe yourself to be fat when others say you are too thin? |
|  | SCOFF-5 | Would you say that food dominates your life? |
| SASS | SASS-1 | It takes me time to overcome my shyness in new situations. |
|  | SASS-2 | I have trouble working when someone is watching me. |
|  | SASS-3 | I get embarrassed very easily. |
|  | SASS-4 | I don't find it hard to talk to strangers. |
|  | SASS-5 | I feel anxious when I speak in front of a group. |
|  | SASS-6 | Large groups make me nervous. |
| AASBV | AASBV-A | I feel nervous about aspects of my physical appearance. |
|  |  | I worry about how others are evaluating how I look. |
|  |  | I feel uncomfortable with certain aspects of my physical appearance. |
|  |  | I am concerned about my ability to attract romantic partners. |
|  |  | I get nervous when others comment on my appearance. |
|  | AASBV-B | I am comfortable with my appearance. |
|  |  | I like how I look. |
|  |  | I am satisfied with my body’s build or shape. |
|  |  | I feel comfortable with my facial attractiveness. |
|  |  | I am satisfied with my body weight. |
|  |  | I am confident that others see me as physically appealing. |
|  | AASBV-C | I would like to change the way I look. |
|  |  | I feel that most of my friends are more physically attractive than myself. |
|  |  | I wish I were better looking. |

SCOFF, the Sick, Control, One, Fat, Food questionnaire; SASS, the Social Anxiety Subscale of the Self-Consciousness Scale; AASBV, Appearance Anxiety Scale Brief Version.

Table S2. Predictability of each item among three groups.

| **Variable** | **Total** | **Male** | **Female** |
| --- | --- | --- | --- |
| SCOFF | 0.18 | 0.14 | 0.199 |
| SASS-1 | 0.459 | 0.462 | 0.442 |
| SASS-2 | 0.465 | 0.483 | 0.442 |
| SASS-3 | 0.553 | 0.546 | 0.544 |
| SASS-4 | 0.095 | 0.081 | 0.114 |
| SASS-5 | 0.63 | 0.629 | 0.617 |
| SASS-6 | 0.631 | 0.627 | 0.619 |
| AASBV-A | 0.609 | 0.608 | 0.610 |
| AASBV-B | 0.169 | 0.137 | 0.212 |
| AASBV-C | 0.522 | 0.514 | 0.524 |

Note: SCOFF, the Sick, Control, One, Fat, Food questionnaire; SASS, the Social Anxiety Subscale of the Self-Consciousness Scale; AASBV, Appearance Anxiety Scale Brief Version. Predictability indicates how much variation in a node can be predicted by variation in the nodes connected to it.

Figure S1. Bootstrapped confidence intervals of edge weights among three groups

The analysis of the accuracy of edges, as implemented by means of non-parametric CIs, revealed that the precision of edges was acceptable, with smaller CIs indicating more accurate estimation of edges. Cis for each population is pretty small, which means the edges analysis is accurate.

Total Male Female


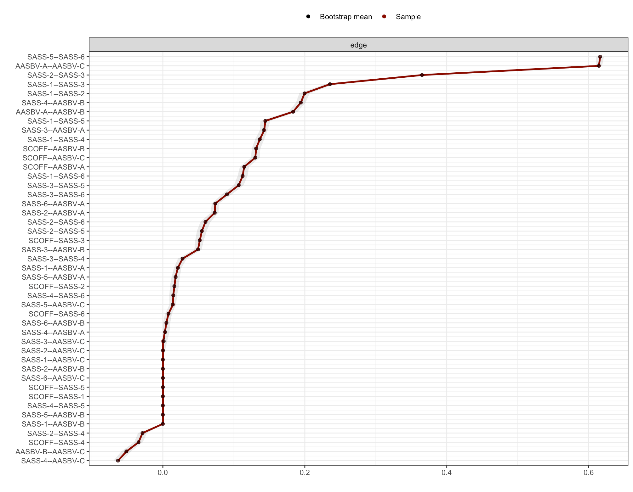

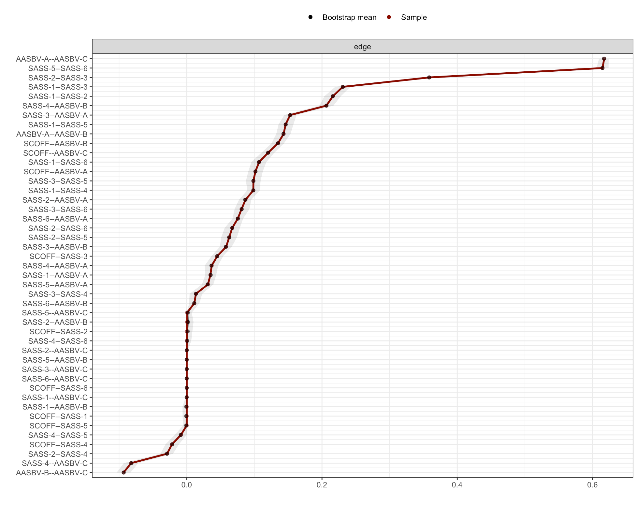

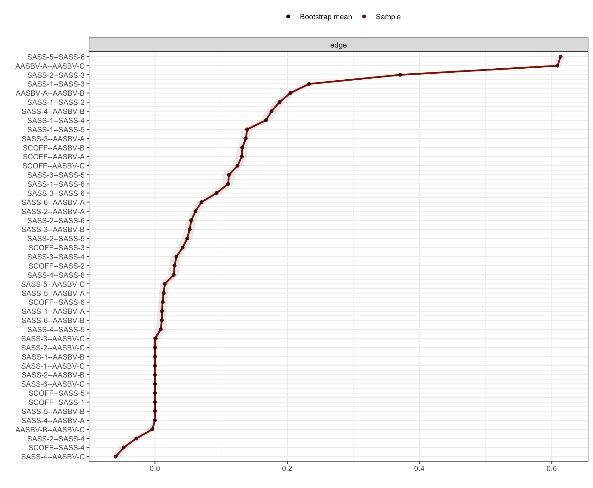


Figure S2. Stability of centrality indices by case dropping subset bootstrap

The case-dropping subset bootstrap procedure showed that the values of betweenness, closeness, and strength remained stable even after dropping large proportions of the sample since the dataset is very large.

Total Male Female


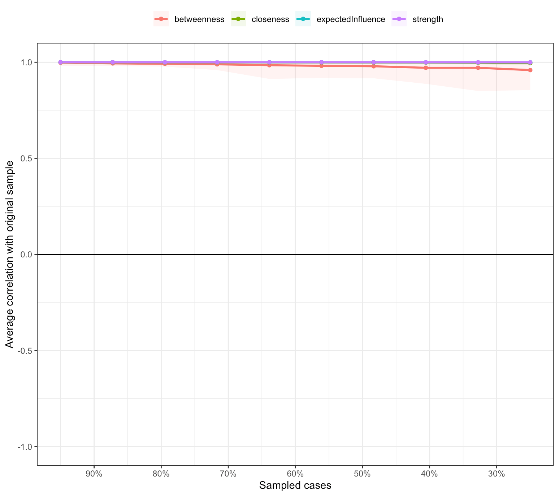

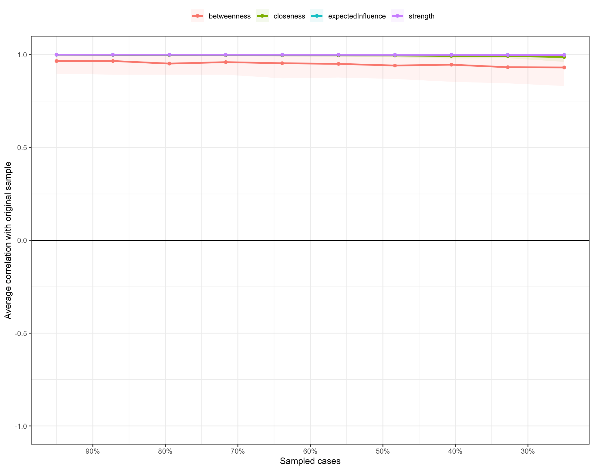

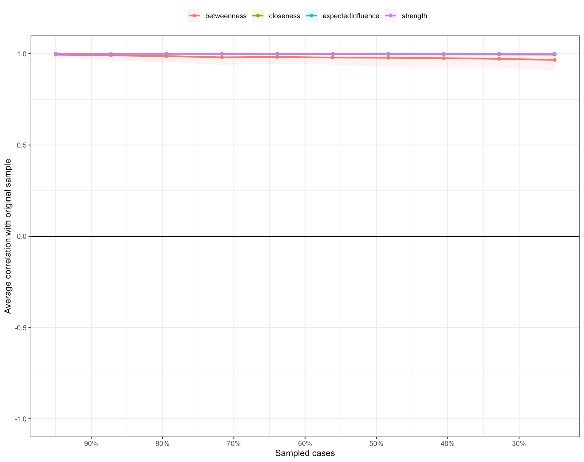


Figure S3. Estimation of "ExpectedInfluence" by bootstrapped difference test among three groups.

The bootstrapped difference tests revealed that a large proportion of the comparisons among edge weights were statistically significant (black means significant, gray means not significant)

Total Male Female


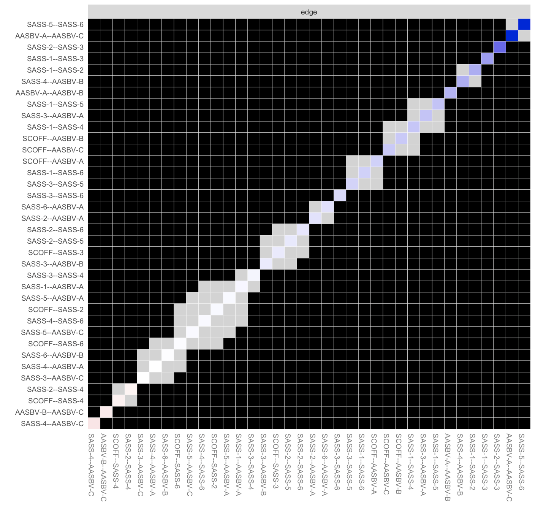

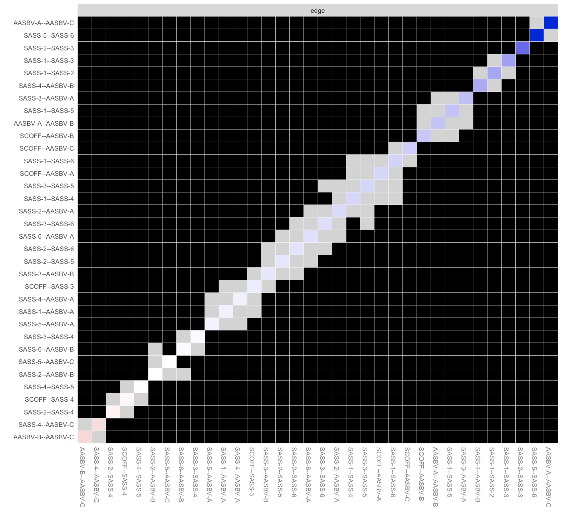

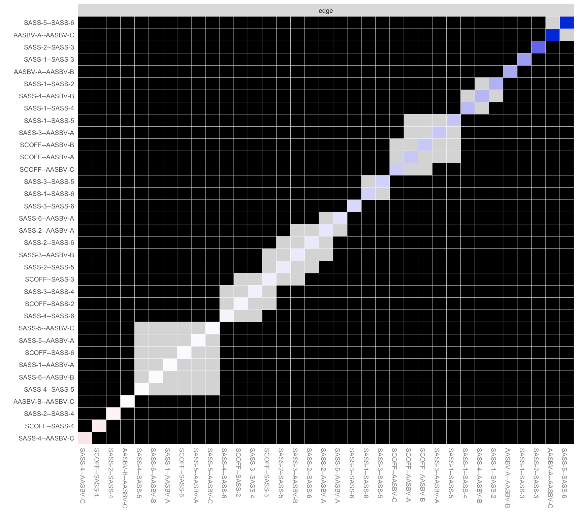


Figure S4. The comparison of network models between males and females.


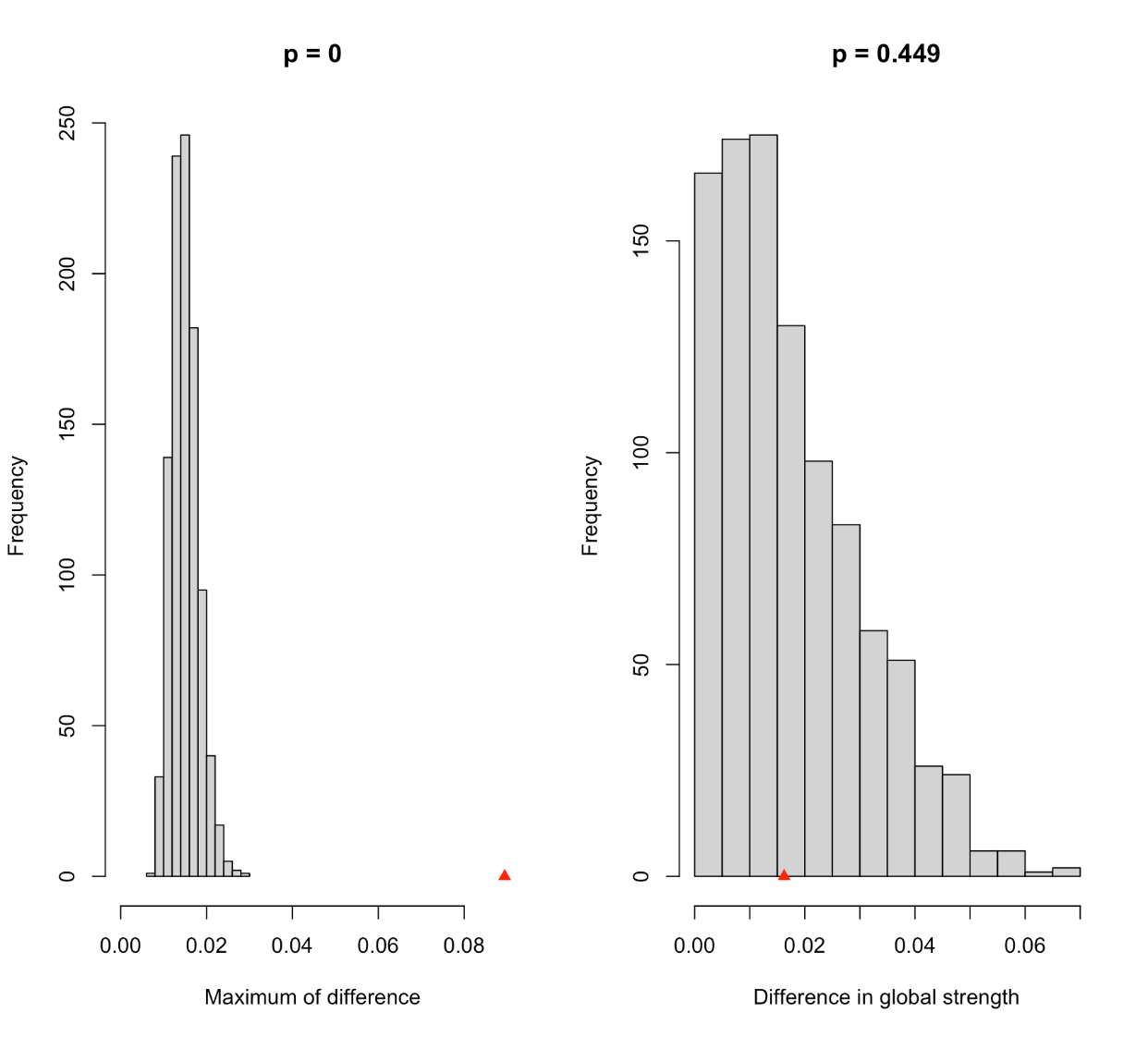


The Network Comparison Test (NCT) is a permutation test to investigate invariance in different network characteristics.

Left Panel: Plot of bootstrap value of the maximum difference in any of the edge weights (1000 permutations), with significant difference (M=0.089, p<0.0001).

Right Panel: Plot of bootstrap value of the difference in network global strength, with significant difference (network strength among male participants: 4.17; among female participants: 4.19; S: 0.016, p = 0.45).

Invariance in edges weights was examined using the permutation test, generating sets of p values for each edge-edge comparison. Holm-Bonferroni corrected p values of 16% edge-edge comparison were <0.05 indicating significant differences. Rest of them has no significant difference.
